# Inflammatory myofibroblastic tumor causing unexplained anemia in a toddler: a case report

**DOI:** 10.1186/1752-1947-5-69

**Published:** 2011-02-17

**Authors:** Mohammad Salameh, Iyad Sultan, Maha Barbar, Maysa Al Hussaini, Abdullah Jameel, Khalil Ghandour, Ma'in Masarweh

**Affiliations:** 1 Department of Surgery, King Hussein Cancer Center, Amman, Jordan; 2 Department of Pediatrics, King Hussein Cancer Center, Amman, Jordan; 3 Department of Pathology, King Hussein Cancer Center, Amman, Jordan; 4 Department of Radiology, King Hussein Cancer Center, Amman, Jordan

## Abstract

**Introduction:**

Inflammatory myofibroblastic tumor is a very rare benign tumor in children that mimics malignant tumors in its aggressiveness locally and by the possibility of recurrence after surgical resection, and causing anemia of chronic disease, which is a decrease in hemoglobin 1 to 2 g/dL below normal level in a patient with chronic illness.

**Case presentation:**

A 32-month-old boy from Libya presented with microcytic hypochromic anemia. He had been treated in three countries and five centers without response to medical therapy. He was investigated at our center and found to have a mass in the colon causing intermittent intussusception and bleeding. He was treated surgically, and his condition improved dramatically. The pathology report proved a diagnosis of inflammatory myofibroblastic tumor.

**Conclusion:**

We report a case of an unusual tumor of the gastrointestinal tract causing chronic anemia not responding to medical treatment, and discuss the characteristics of inflammatory myofibroblastic tumor. In our case, we stress the involvement of a multidisciplinary team in treating such a patient who presents with common symptoms and signs but in whom there has been no response to any of the measures and treatment protocols.

## Introduction

Anemia of chronic disease is an entity that explains a decrease in hemoglobin, usually 1 to 2 g/dL below normal level in a patient with a chronic illness. This form of anemia is multifactorial and can be caused by autoimmune diseases, chronic infections or neoplasms. Erythrocytes are usually normocytic but can be microcytic, in which case distinguishing it from iron-deficiency anemia can be challenging. When anemia of chronic disease is suspected, a systematic approach to explore possible causes should be used to avoid unnecessary investigations and to establish the diagnosis.

Inflammatory myofibroblastic tumor (IMT) is a very rare benign tumor that mimics malignant tumors in its aggressiveness locally and by the possibility of recurrence after surgical resection. Grossly, the tumor is a solid, well-circumscribed, nonencapsulated mass that could be found in many anatomic locations, including the colon, lung, bladder, spleen, breast, pancreas, liver, spermatic cord, prostate, peripheral nerves, soft tissue and orbit. The whole mark of these tumors is the spindle cell proliferation, which are in fact myofibroblasts, associated with a variable inflammatory component and thence the name [1]. Herein we report a case of IMT that arises from the transverse colon causing colocolic intussusception, and we discuss the importance of a multidisciplinary approach to reach a diagnosis in such cases.

## Case presentation

A 32-month-old boy from Libya presented with a picture of microcytic hypochromic anemia. He was previously healthy until the age of 16 months, when he was found to have pallor, loss of appetite and hypoactivity. The patient was diagnosed with iron-deficiency anemia (hemoglobin, 7 mg/dL) and started on iron supplements with no response to treatment. He underwent extensive investigations in six different institutions and three different countries. He received five blood transfusions and was kept on oral ferrous sulphate.

The patient was started on a gluten-free diet on the assumption of celiac disease, but blood test and endoscopic biopsy results of the upper gastrointestinal (GI) tract were normal. He was also treated with metronidazole because of amoebic cysts in the stool. Two weeks before referral, the patient was diagnosed with Coomb's negative autoimmune hemolytic anemia and was started on prednisone (2 mg/kg/day) with a dramatic response as his hemoglobin increased from 7.0 g/dL to 9.3 g/dL and reticulocytes increased from 1% to 9%. At our institution, the patient's history and medical record were thoroughly reviewed. He had a history of intermittent abdominal pain associated with chronic constipation treated with laxatives. There were episodes of mucus in the stool associated with streaks of blood. On examination, the child was irritable with facial swelling and abdominal distension. He looked pale with no jaundice. He had clubbing of the fingers. He had normal air entry. Heart auscultation revealed a soft systolic murmur. He had no lymphadenopathy and no hepatosplenomegaly. There were no abdominal masses appreciated on the first visit, but on a later visit, a left side and suprapubic mass was felt, and a hydrocele was found upon scrotal examination. During this time, prednisone was tapered and stopped.

**Figure 1:**
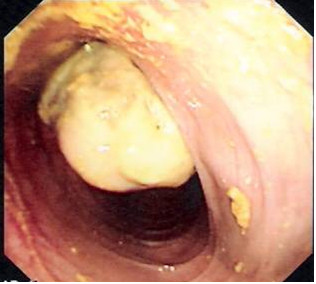
**Colonoscopic view**.

Lower GI endoscopy was performed on the assumption of inflammatory bowel disease; the mucosa of the rectum and sigmoid were normal, but there was a mass in the sigmoid covered by a layer of exudate, and the scope could not go beyond this mass (Figure 1). Lower GI contrast study was carried out to delineate the proximal colon, but the picture was suggestive of intussusception.

**Figure 2:**
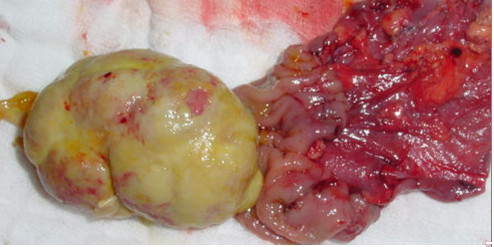
**Intraoperative view**.

A computed tomography scan of the abdomen showed a mass in the left side of the abdomen with suspicion of intussusception. Ultrasonography was not helpful in identifying the nature of that mass. Laparotomy was done and showed that there was an intraluminal mass reaching the sigmoid colon originating from the mid transverse colon, causing colocolic intussusception (Figure 2). Resection of the involved segment of the colon was done with end-to-end anastomosis. The patient had a smooth postoperative course, and his hemoglobin level improved dramatically and quickly during the first week after surgery. The histopathology showed extensive surface ulceration and was characterized by a proliferation of plump polygonal or more spindle-shaped cells with amphophilic cytoplasm and vesicular nuclei with prominent nucleoli. The lesion cells were distributed in a patternless fashion within a loose myxoid matrix, which contained numerous thin-walled blood vessels and prominent mixed inflammatory cells, including numerous plasma cells and lymphocytes.

Immunostains showed positivity for S100, keratin AE1/AE3, and more focally for desmin. Smooth muscle actin and anaplastic lymphoma kinase 1 were negative.

## Discussion

IMTs are benign solid tumors that arise in many anatomic sites. The lung is the most common site, but other sites are also reported, including the small and large bowel mesentery, mediastinum, retroperitoneum, omentum, spleen, spermatic cord, prostate, peripheral nerves, soft tissue, orbit and diaphragm [1-3]. IMT is considered a benign tumor with local aggressive course but is not a malignancy in itself.

The presentation of IMT varies depending on the site and includes fever, weakness, abdominal pain, upper GI bleeding, weight loss, vomiting, poor appetite, gastroesophageal reflux, pallor, growth retardation and ascites. These symptoms can appear either alone or in a combination [4]. Laboratory investigations are not specific. The most common finding is microcytic hypochromic anemia refractory to iron therapy (90.9%), but other laboratory findings, such as a high erythrocyte sedimentation rate, thrombocytosis, eosinophilia and hypergammaglobulinemia, may be seen [4,5].

Histopathologically, IMT is characterized by spindle-shaped cells with chronic inflammatory cell infiltrate consisting of plasma cells, lymphocytes and occasional histiocytes [1,6]. These inflammatory cells are usually scattered throughout the lesion to a variable degree [1]. Three different histologic patterns have been described. In the first, myofibroblasts have a spindle appearance and are distributed loosely in a myxoid stroma. The second is more densely packed stromal cells intermingled with an inflammatory component. The third is characterized by hyalinized, hypocellular stroma. These three patterns can be found in a single tumor intermixed closely [1]. All of these features resolve after resection of the tumor [5]. Karnak *et al. *[5] reported a case with very high leukocyte count and suggest that this could be a good marker for recurrence. Differentiating IMT from sarcoma may be challenging because of the local invasiveness of both pathologies. The lack of mitosis and nuclear atypia in IMT can differentiate it from sarcomas [7].

About 18% to 40% of IMTs recur, and most recurrences appear in extrapulmonary lesions that are larger than 8 cm and are locally invasive [6,8]. Retroperitoneal and mesenteric IMT seem to be associated with more frequent recurrences [9]. Complete surgical resection is the treatment of choice and should be advocated unless prohibited [1,9].

Controversy still exists regarding the role of chemotherapy in IMT patients. According to Kovach *et al. *[1], chemotherapy has been reserved for patients for whom resection is neither complete nor possible. The dosage and regimen of chemotherapy should be adjusted according to the biologic aggressiveness of the tumor, and there is no good evidence to support chemotherapy after complete resection regardless of tumor biology [1]. Radiation treatment showed some benefit in pulmonary IMT [10]. On the other hand, failure of radiation therapy in treating IMT has been reported, confirming surgical excision to be the treatment of choice [11]. Radiation therapy is used in palliative treatment of this tumor, decreasing the mass effect of IMT and in patients whose tumors are not resectable [1]. The use of steroids is also recommended to reduce the inflammatory process that is surrounding the tumor, especially if the tumor is in the central nervous system [1,12]. Using nonsteroidal antiinflammatory drugs as a conservative measure for treatment of patients with IMT whose tumors are not amenable to surgical resection [13].

## Conclusion

In our case, we stress on the involvement of multidisciplinary team in treating such a patient who presents with common symptoms and signs but who has not responded to the measures and treatment protocols; this team should involve medical, surgical, radiology and pathology personnel.

## Consent

Written informed consent was obtained from the patient's father for publication of this case report and accompanying images. A copy of the written consent is available for review by the Editor-in-Chief of this journal.

## Competing interests

The authors declare that they have no competing interests.

## Authors' contributions

MS was a major contributor in writing the manuscript. IS was the oncologist who took care of the child during his diagnosis and treatment. MB was the gastroenterologist who performed the endoscopy. MAH was the pathologist who reviewed the pathology specimen. AJ was the radiologist involved in the radiologic diagnostic tests. KG was the surgeon who performed the surgical procedure. IS, MB, MAH, AJ and KG also reviewed and contributed to the writing of the manuscript. MM was the surgeon who performed the surgery and was a major contributor in writing the manuscript. All authors read and approved the final manuscript.

## References

[B1] Kovach SJ Fischer AC Katzman PJ Salloum RM Ettinghausen SE Madeb R Koniaris LG Inflammatory myofibroblastic tumors J Surg Oncol 2006 94 5 385 391 10.1002/jso.20516 16967468

[B2] Souid AK Ziemba MC Dubansky AS Mazur M Oliphant M Thomas FD Ratner M Sadowitz PD Inflammatory myofibroblastic tumor in children Cancer 1993 72 6 2042 2048 10.1002/1097-0142(19930915)72:6<2042::AID-CNCR2820720641>3.0.CO;2-I 8364883

[B3] Cviko A Milic Z Cizmic A Seiwerth S Kruslin B Inflammatory myofibroblastic tumor with extensive involvement of the bowel in a 7-year-old child Croat Med J 1999 40 4 550 553 10554359

[B4] Cho MY Min YK Kim NR Cho SJ Kim HK Lee KC Suh SO Whang CW Fever of unknown origin as a presentation of gastric inflammatory myofibroblastic tumor in a two-year-old boy J Korean Med Sci 2002 17 5 699 703 12378027 10.3346/jkms.2002.17.5.699 PMC3054930

[B5] Karnak I Senocak ME Ciftci AO Cağlar M Bingöl-Koloğlu M Tanyel FC Büyükpamukçu N Inflammatory myofibroblastic tumor in children: diagnosis and treatment J Pediatr Surg 2001 36 6 908 912 10.1053/jpsu.2001.23970 11381424

[B6] Coffin CM Watterson J Priest JR Dehner LP Extrapulmonary inflammatory myofibroblastic tumor (inflammatory pseudotumor): a clinical and immunohistochemical study of 84 cases Am J Surg Pathol 1995 19 8 859 872 10.1097/00000478-199508000-00001 7611533

[B7] Sanders BM West KW Gingalewski C Engum S Davis M Grosfeld JL Inflammatory pseudotumor of the alimentary tract: clinical and surgical experience J Pediatr Surg 2001 36 1 169 173 10.1053/jpsu.2001.20045 11150459

[B8] Dao AH Hodges KB Inflammatory pseudotumor of the pelvis: case report with review of recent developments Am Surg 1998 64 12 1188 1191 9843343

[B9] Huang CC Lien HH Chen DF Tsai MS Pediatric intra-abdominal inflammatory myofibroblastic tumor Asian J Surg 2006 29 1 58 61 10.1016/S1015-9584(09)60299-2 16428104

[B10] Hoover SV Granston AS Koch DF Hudson TR Plasma cell granuloma of the lung, response to radiation therapy: report of a single case Cancer 1977 39 1 123 125 10.1002/1097-0142(197701)39:1<123::AID-CNCR2820390121>3.0.CO;2-H 401674

[B11] Mehta J Desphande S Stauffer JL Stanford R Fernandez E Plasma cell granuloma of the lung: endobronchial presentation and absence of response to radiation therapy South Med J 1980 73 9 1198 1201 7414377 10.1097/00007611-198009000-00009

[B12] Chun YS Wang L Nascimento AG Moir CR Rodeberg DA Pediatric Inflammatory myofibroblastic tumor: anaplastic lymphoma kinase (ALK) expression and prognosis Pediatr Blood Cancer 2005 45 6 796 801 10.1002/pbc.20294 15602716

[B13] Su W Ko A O'Connell T Applebaum H Treatment of pseudotumors with nonsteroidal anti-inflammatory drugs J Pediatr Surg 2000 35 11 1635 1637 10.1053/jpsu.2000.18340 11083441

